# Naringin mitigates testicular injury and associated neuronal toxicity in lead-exposed cockerel chicks

**DOI:** 10.22038/AJP.2023.22946

**Published:** 2024

**Authors:** Oluwaseun Olanrewaju Esan, Chinomso Gift Ebirim, Moses Olusola Adetona, Temitayo Olabisi Ajibade, Ademola Adetokunbo Oyagbemi, Temidayo Olutayo Omobowale, Omolade Abodunrin Oladele, Adeolu Alex Adedapo, Momoh Audu Yakubu, Evaristus Nwulia, Oluwafemi Omoniyi Oguntibeju

**Affiliations:** 1 *Department of Veterinary Medicine, Faculty of Veterinary Medicine, University of Ibadan, Nigeria*; 2 *Department of Anatomy, Faculty of Basic Medical Sciences, University of Ibadan, Nigeria*; 3 *Department of Veterinary Physiology and Biochemistry, Faculty of Veterinary Medicine, University of Ibadan, Nigeria*; 4 *Department of Veterinary Pharmacology and Toxicology, Faculty of Veterinary Medicine, University of Ibadan, Nigeria*; 5 *Department of Environmental & Interdisciplinary Sciences, College of Science, Engineering & Technology, COPHS, Texas Southern University, Houston, TX, USA*; 6 *Howard University, College of Medicine, Department of Psychiatry and Behavioral Sciences, Howard University Hospital, 2041 Georgia Avenue, Washington, DC 20060, USA*; 7 *Department of Biomedical Sciences, Faculty of Health and Wellness Sciences, Cape Peninsula University of Technology, Bellville 7535, South Africa*

**Keywords:** Lead toxicity, Oxidative stress, Testicular apoptosis Neuroinflammation

## Abstract

**Objective:**

Lead (Pb) poisoning affects multiple organs including the reproductive system. The experiment was performed to explore the protective effect of naringin on testicular apoptosis, neuronal dysfunction and markers of stress in cockerel chicks.

**Materials and Methods:**

Thirty-six cockerel chicks were used for this study, and randomly grouped into six chicks per group viz. control, Pb only (600 ppm), Pb and naringin (80 mg/kg), Pb and Naringin (160 mg/kg), naringin only (80 mg/kg) and naringin only (160 mg/kg), respectively, for eight weeks. Pb was administered via drinking water while naringin was administered via oral gavage. Oxidative stress indices in the brain and testes were assessed, and immunohistochemistry of TNF-α and caspase 3 was done in the brain and testes, respectively.

**Results:**

Lead administration induced inflammatory and testicular apoptosis cascade accompanied with increased oxidative stress and upregulation of brain and testicular antioxidant enzymes in comparison to the control and Pb-only-treated cockerels. Immunohistochemistry showed significant immunoreactivity of testicular caspase 3 and TNF-α in the brain.

**Conclusion:**

Treatment of Pb-exposed chickens with naringin offered protection to Pb acetate-induced testicular oxidative stress, apoptosis, and neuroinflammation in cockerel chicks.

## Introduction

Lead (Pb) is a common, non-biodegradable contaminant that has a significant impact on human and animal health. Pb is naturally found in small amounts, nevertheless, industrial activities have been reported to considerably increase the concentration of Pb (Al-Megrin et al., 2020). Human exposure to Pb can be through ingestion and inhalation. Pb toxicity is very harmful on people, animals, and plants (Yousef et al., 2019). Pb poisoning is induced by free radical generation and inhibition of antioxidant enzymes. Reactive oxygen species (ROS) can chelate atoms of oxygen and nitrogen causing numerous enzymes and proteins to malfunction (Abdel Moneim et al., 2011). Oxidative insults also affect DNA structure and prevent damaged DNA from being repaired, which can lead to aberrant gene expression.

Pb has also been discovered to elicit inflammation by promoting immune and non-immune cells, causing them to over-secrete inflammatory mediators, which may drive apoptotic signaling pathway (Chen et al., 2019). Reproductive organs are extremely susceptible to stressful situations and can be damaged by any hazardous element with Pb having a direct impact on testicular shape (Khodabandeh et al., 2021). Heavy metals have been reported to inhibit enzymatic function and impact sperm properties such as motility, morphology, and concentration (El-Khadragy et al., 2020). Pb is thought to have an indirect effect on male reproductive function through disruption of the endocrine system (Abiodun and Adeleye, 2020). It also causes spermatozoa death and genetic instability as well as activating apoptosis in spermatogenic cells. It also inhibits spermatogenic activity by lowering sperm calcium levels and decreasing sperm protein phosphorylation. Lead exposure has been reported to have a negative effect on hormone secretion by suppressing luteinizing hormone and follicle stimulating secretion from the anterior pituitary (El-Khadragy et al., 2020; Kumar and Devi, 2018). 

Umbelliferone has been found to prevent testicular injury induced by Pb toxicity via suppressing oxidative damage, inflammation, and cell death, and boosting antioxidant defenses, Nrf2/HO-1 signaling and pituitary-gonadal axis (Alotaibi et al., 2020). Khodabandeh et al. (2021) reported that quercetin preserved testes architecture and mitigated apoptosis against Pb-acetate toxicity. In another study, Olaniyan et al. (2021) documented that *Cocos nucifera *L. oil alleviates lead acetate-induced reproductive toxicity in sexually matured male Wistar rats. The amino acid L-carnitine was reported to ameliorate chronic toxicity associated with Pb toxicity (Abdel-Emam and Ahmed, 2021). Interestingly, Ibrahim et al. (2021) demonstrated that *Chlorella vulgaris* and *Spirulina platensis* mitigated Pb acetate-induced testicular oxidative stress and apoptosis, thereby corroborating the ealier report of Khodabandeh et al. (2021). Again, *Allium triquetrum* was found to significantly alleviate Pb acetate-induced reproductive injuries by boosting sperm characteristics and ameliorating oxidative stress markers (Kahalerras et al., 2022). The antioxidative, anti-inflammatory and anti-apoptotic actions of ellagic acid, resveratrol, and *Asparagus officinalis* against Pb acetate-induced testicular and hepato-renal oxidative damages have also been reported (Bidanchi et al., 2022; Mitra et al., 2022; Alyami et al., 2022). Recently, Bentaiba et al. (2023) reported that *Withania frutescens* root extract restored testicular damage induced by Pb toxicity.

The damage to the central nervous system (CNS) induced by Pb pollution requires immediate and urgent attention. Pb exposure has been linked to behavioral abnormalities as well as cognitive and mental development deficiencies in a growing number of studies (Ogundele, 2018). Furthermore, in human and animal studies, Pb exposure could cause emotional abnormalities or even neuropsychiatric problems (Zhang et al., 2021). Alterations in neurotransmitter system and synaptic signaling, mitochondrial malfunction, and oxidative stress, are all plausible pathways for Pb neurotoxicity, with oxidative stress being a focus of attention (Zhang et al., 2021). ROS are frequently involved in negative effects of oxidative, exaggerated increase in free radicals’ generation and reduction in the anti-oxidative status (Ahmad et al., 2018). Pb is known to compete with Fe^2+^, and the buildup of free iron causes oxidative stress and neurotoxicity as reported by Ferreira et al. (2017).

The nervous system is particularly sensitive to oxidative stress due to the presence of high metabolic activity, inadequate antioxidant systems, and fatty acid content (Yu et al., 2021). Pb as a neurotoxin interferes with and diminishes ion calcium communication in nerve processes, thereby affecting the cerebral cortex, cerebellum, and hippocampus. There has been a positive correlation between peripheral nerve degeneration and apoptosis in the neural tissue of encephalopathy induced by Pb intoxication (Al-Khafaf et al., 2021). 

Naringin (NAR) is a polyphenolic chemical compound found in high concentration in citrus and grapefruits. In experimental trials, it has been shown to have several therapeutic effects against a variety of diseases, including neurodegenerative disorders (Varshney and Garabadu, 2021). In *in-vivo* and *in-vitro* experimental models, NAR was documented to possess antioxidant property (Qin et al., 2016). In numerous animal models, NAR has been shown to improve memory development (de-Andrade Teles et al., 2016). In the animal brain, it has been shown to promote cholinergic function while inhibiting acetylcholinesterase activity (Varshney and Garabadu, 2021).

In a cell culture investigation, it was discovered that NAR improves mitochondrial activity and apoptosis, possibly through heme oxygenase-1 (HO-1)-mediated action (Varshney and Garabadu, 2021). NAR pretreatment has also been shown to increase learning and memory formation in rats (Ghofrani et al., 2015). Recently, it was shown that NAR has therapeutic effects in experimental animals via the renin-angiotensin system (RAS) as previously described by Wang et al. (2019). NAR has also been reported to improve neuroprotection by lowering oxidative stress and enhancing neuronal survival (Gindri et al., 2021). NAR has been documented to undergo enterohepatic biotransformation to release naringenin which can cross the blood brain barrier (BBB) and trigger a variety of protective activities (Wang et al., 2019). 

In this work, our aim was to understand the protective actions of NAR on the reproductive and central nervous systems and explore its possible molecular mechanism of action. We also explored the metal-chelating antioxidant activity of NAR on the toxicity of Pb. We hypothesized that the inclusion of NAR in poultry feeds might be beneficial as an antioxidant with metal binding capacity. 

## Materials and Methods

**Chemicals**

Pb acetate trihydrate ((C₂H₃O₂)2Pb.3H₂O); CAS No.: 6080-56-4, naringin; CAS No.: 6751AF, Odianisidine; CAS No.: 119-90-4, H2O2; CAS No.: 7722-84-1, xylenol orange (XO); CAS No.: F943, potassium hydroxide; CAS No.: 1993AH, GSH; CAS No.: 70-18-8, oxidized glutathione (GSSG); CAS No.: 27025-41-8, thiobarbituric acid (TBA); CAS No.: 504-17-6, 1,2dichloro4nitrobenzene; CAS No.: 99-54-7, from Sigma (St. Louis, MO, USA). Normal goat serum, Biotinylated, and antibody 2-step plus Poly-HRP Anti Mouse/Rabbit IgG Detection System with DAB (E-IR-R217) solution were purchased from Elabscience Biotechnology®, China), anti-tumor necrosis factor (TNF-α) polyclonal antibody (E-AB-63550: 1:500 Dilution) for brain and caspase 3 monoclonal antibody (E-AB-22213: 1:500 Dilution) for the testes. 

**Animals housing and experimental design**

Thirty-six (36) day old cockerel chickens aged ten weeks were distributed into six groups of six chickens each. Group A served as the Control, Group B (Pb; 600 parts per million; ppm), Group C (Pb and 80 mg/kg Naringin), Group D (Pb and 160 mg/kg Naringin), Group E (80 mg/kg Naringin) and Group F (160 mg/kg Naringin). Pb administration was done based on previously reported studies (Wang et al., 2012; Oladapo et al., 2021). The chicks were liberally supplied poultry feed from Top Feeds Limited, Ibadan, and water *ad libitum*. The vehicle used for this work was distilled water for Pb and NAR. Pb and NAR were administered orally. The study was approved with UI-ACUREC/ 021-0421/16 approval number.

**Preparation of testes and brain cytosolic fractions**

For the preparation of tissue homogenates, the brain and testes were quickly excised, rinsed, blotted with filter paper, weighed, chopped into bits, and homogenized with homogenizing buffer (0.1 M phosphate buffer, pH 7.4) using a Teflon homogenizer for thirty strokes each. The resulting homogenate was centrifuged at 10,000 x *g* for 10 min with a cold centrifuge at -4˚C to obtain post mitochondrial fractions (PMFs). The supernatants (PMFs) were used for biochemical assays.

**Determination of** ***in vivo*** **antioxidant status**

The superoxide dismutase (SOD) assay was carried out by the method of Oyagbemi et al. (2015). Briefly, 100 mg of epinephrine was dissolved in 100 ml distilled water and acidified with 0.5 ml concentrated hydrochloric acid. Thirty microliters of PMF were added to 2.5 ml 0.05 M carbonate buffer (pH 10.2) followed by the addition of 300 ml of 0.3 mM adrenaline. The increase in absorbance at 480 nm was monitored every 30 sec for 150 sec. The one unit of SOD activity was given as the amount of SOD necessary to cause 50% inhibition of the autooxidation of adrenaline to adrenochrome. Beutler et al. (1963) study was used to determine glutathione peroxidase (GPx), and glutathione S-transferase (GST) was estimated by the method of Habig et al. (1974) and Beutler et al. (1963). The reaction mixtures contained 0.5 ml of potassium phosphate buffer (pH 7.4), 0.1 ml of sodium azide 0.2 ml of GSH solution, 0.1 ml of H_2_O_2_, 0.5 ml of sample and 0.6 ml of distilled water. The mixture was incubated in a water bath at 37^o^C for 5 min and 0.5 ml of trichloroacetic acid was added and centrifuged at 4,000 rpm for 5 min. A volume of 1 ml of the supernatant was taken and added 2 ml of K_2_PHO_4_ and 1 ml of Ellman's reagent. The absorbance was read at 412 nm using distilled water as blank. The reduced glutathione (GSH) content was estimated by an established method (Salbitani et al., 2017). Here, 0.5 ml of 4% sulfo- salicylic acid (precipitating agent) was added to 0.5 ml of sample and centrifuged at 4,000 rpm for 5 min. To 0.5 ml of the resulting supernatant, 4.5 ml of Ellman's reagent (0.04 g of DTNB in 100 ml of 0.1 M phosphate buffer, pH 7.4) was added. The absorbance was read at 412 nm against distilled water as blank. 

**Determination of oxidative stress indices**

The content of H_2_O_2_ generated was quantified by Wolff’s method (Wolff, 1994). To 50 μl of sample, 2.5 ml 0.1M potassium phosphate buffer (pH 7.4), 250 μl of ammonium ferrous sulfate, 100 μl of sorbitol, 100 μl of xanthine oxidase and 25 μl of H_2_SO_4_ were added and the mixture was thoroughly vortexed until a light pink color of the reaction mixture was observed. The reaction mixture was subsequently incubated at room temperature for 30 min. The optical density was read at absorbance at 560 nm. The H_2_O_2_ generated was extrapolated from H_2_O_2 _standard curve. 

Malondialdehyde (MDA) content was determined following the protocol of Varshney and Kale (1990). Malondialdehyde (MDA), a lipid peroxidation by-product was quantified in the PMFs of neural and testicular tissues according to the method of Varshney and Kale (1990). To 1.6 ml of Tris-KCl, 0.5 ml of 30% TCA, 0.4 ml of sample, and 0.5 ml of 0.75% thiobarituric acid prepared in 0.2 M HCl were added and mixed well. The reaction mixture was incubated in a water bath at 80^°^C for 45 min to generate a pink colored malondialdehyde and centrifuged at 4,000 rpm for 15 min. The absorbance was measured against a blank at 532 nm. Lipid peroxidation levels were extrapolated with a molar extinction coefficient of 1.56×10^5^/M/cm. 

**Protein concentration and nitric oxide content**

Protein contents were determined by the method of Wilson and Walker (2010). Mixture was incubated at room temperature for 30 min, and values were read with spectrophotometer at 540 nm using distilled water as blank. The final value for total protein was extrapolated from total protein standard curve using Bovine Serum Albumin. 

**Histopathology**

Testes and brain were fixed in 10% formalin and Bouin’s solution, respectively, embedded in paraffin wax, and sections of 5-6 um in thickness were made and thereafter stained with Haematoxylin and Eosin (H&E) as previously described by Rotimi et al. (2000).

**Immunohistochemistry**

Immunostaining was performed as earlier described for caspase 3 monoclonal antibody in the testis and TNF-α polyclonal antibody in the brain tissues **(**Oyagbemi et al., 2022). Sections were observed with light microscope (Leica LAS-EZ®) using Leica software application suite version 3.4 equipped with a digital camera. 

**Data analysis**

One-way analysis of variance (ANOVA) was used to test statistical significance, and the results are expressed as mean±standard deviation (SD). Tukey post-hoc test was used to test for the difference in the variances among different groups. Significant difference between the means of two groups was compared by the student’s t test.

## Results

**O** **xidative stress indices and antioxidant status**

**Figure 1:**
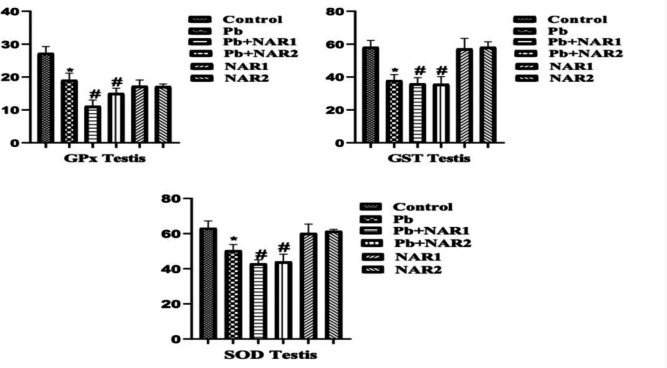
Testicular antioxidant defense status: Superscript (*) indicates significant difference at p<0.05 when compared with Group A (Control), while superscript (#) indicates significant difference when compared with Group B (Pb alone). Group A: Control, Group B: Pb (600 ppm), Group C: Pb and Naringin (80 mg/kg), Group D: Pb and Naringin (160 mg/kg), Group E: Naringin (80 mg/kg), and Group F: Naringin (160 mg/kg). Sample size (n = 6). Abbreviations: GPx; Glutathione peroxidase, GST; Glutathione S-transferase, SOD; Superoxide dismutase; Pb= lead acetate; Pb+NAR1=lead acetate plus naringin 80 mg/kg; Pb+NAR2= lead acetate plus naringin 160 mg/kg; NAR 1= 80 mg/kg; NAR2= 160 mg/kg.

**Figure 2:**
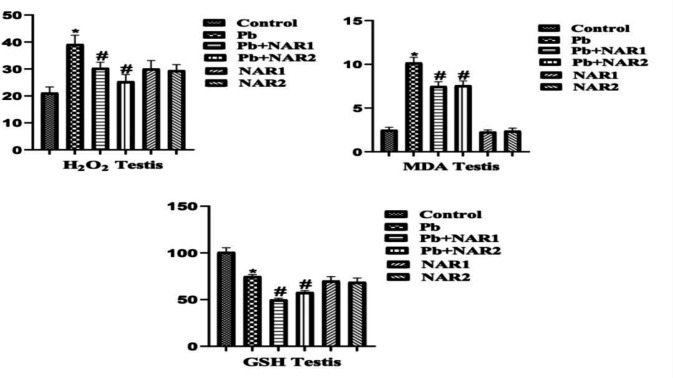
Testicular markers of oxidative stress: Superscript (*) indicates significant difference at p<0.05 when compared with Group A (Control), while superscript (#) indicates significant difference when compared with Group B (Pb alone). Group A: Control, Group B: Pb (600 ppm), Group C: Pb and Naringin (80 mg/kg), Group D: Pb and Naringin (160 mg/kg), Group E: Naringin (80 mg/kg), and Group F: Naringin (160 mg/kg). Sample size (n = 6). Abbreviations: H2O2; hydrogen peroxide, MDA; Malondialdehyde, GSH; Reduced glutathione; Pb= lead acetate; Pb+NAR1=lead acetate plus naringin 80 mg/kg; Pb+NAR2= lead acetate plus naringin 160 mg/kg; NAR 1= 80 mg/kg; NAR2= 160 mg/kg.

**Figure 3:**
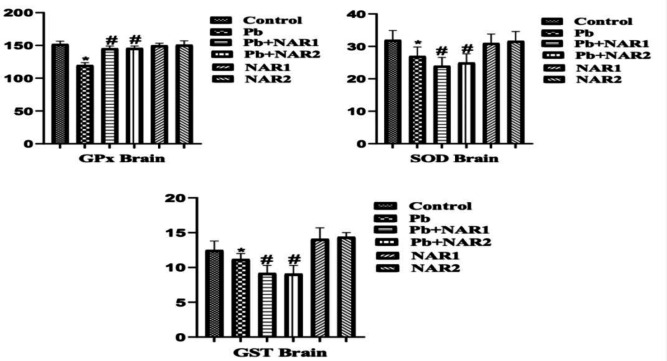
Brain antioxidant defense status: Superscript (*) indicates significant difference at p<0.05 when compared with Group A (Control), while superscript (#) indicates significant difference when compared with Group B (Pb alone). Group A: Control, Group B: Pb (600 ppm), Group C: Pb and Naringin (80 mg/kg), Group D: Pb and Naringin (160 mg/kg), Group E: Naringin (80 mg/kg), and Group F: Naringin (160 mg/kg). Sample size (n = 6). Abbreviations: GPx; Glutathione peroxidase, GST; Glutathione S-transferase, SOD; Superoxide dismutase; Pb= lead acetate; Pb+NAR1=lead acetate plus naringin 80 mg/kg; Pb+NAR2= lead acetate plus naringin 160 mg/kg; NAR 1= 80 mg/kg; NAR2= 160 mg/kg.

**Figure 4:**
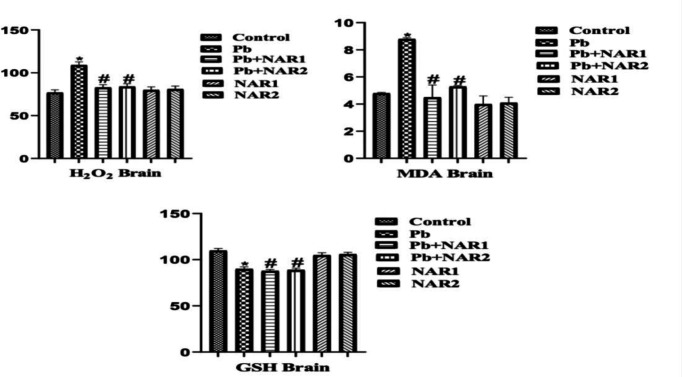
Brain markers of oxidative stress: Superscript (*) indicates significant difference at p<0.05 when compared with Group A (Control), while superscript (#) indicates significant difference when compared with Group B (Pb alone). Group A: Control, Group B: Pb (600 ppm), Group C: Pb and Naringin (80 mg/kg), Group D: Pb and Naringin (160 mg/kg), Group E: Naringin (80 mg/kg), and Group F: Naringin (160 mg/kg). Sample size (n = 6). Abbreviations: H2O2; hydrogen peroxide, MDA; Malondialdehyde, GSH; Reduced glutathione; Pb= lead acetate; Pb+NAR1=lead acetate plus naringin 80 mg/kg; Pb+NAR2= lead acetate plus naringin 160 mg/kg; NAR 1= 80 mg/kg; NAR2= 160 mg/kg.

The results from this study indicated a statistically significant (p<0.05) increase in the activities of testicular GPx, GST, and SOD in chicks intoxicated with Pb compared to the control. The recorded high testicular antioxidant enzymes could be linked to toxicant-induced adaptive response. Co-treatment with NAR however improved testicular antioxidant status (Figure 1). In Figure 2, indices of testicular oxidative stress (H₂O₂ and MDA) are noticed to increase in Pb-exposed chicks compared to the untreated animals. Also, significant reduction in values of MDA was observed in the NAR co-treated with Pb acetate groups (Figure 2). In another experiment, a noticeable lower activity of GPx in the brain, however, increased activities of GST, and SOD of Pb-intoxicated chicks were observed in comparison to the control. NAR co-treatment caused observable improvement in the brain antioxidant activities (Figure 3). We observed higher values in the activities of SOD and GST, and this could be associated with adaptive response. Again, Pb administration enhanced the production of markers of oxidative stress together with depletion of GSH content in comparison to the control and chicks treated with NAR as observed in Figure 4. The metal-chelating and antioxidant power of NAR was demonstrated by reduction in neuronal H_2_O_2_ generation and MDA content as well as appreciable improvement in GSH content (Figure 4).

**Histopathology**

**Figure 5:**
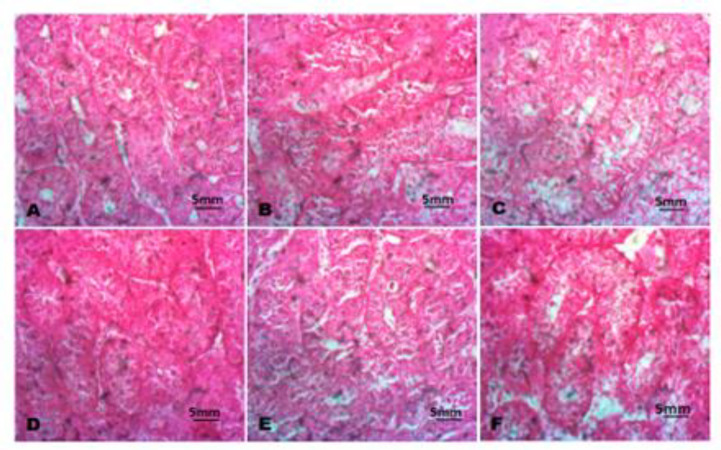
The histopathology of the testes. A (Control), B (Lead acetate; 600 ppm), C (Lead acetate + Naringin 80 mg/kg), D (Lead acetate + Naringin 80 mg/kg), E (Naringin 80 mg/kg), and F (Naringin 100 mg/kg). Slides were stained with Haematoxylin and Eosin. (Magnification x 400).

The histology of the testes revealed Sertoli cells swelling and degeneration of seminiferous tubules in chicks intoxicated with Pb as indicated in Figure 5 compared to the control. Interestingly, cockerel chicks co-administered with Pb and NAR (80 mg/kg), Pb and NAR (160 mg/kg), showed mild swelling of Sertoli cells and degeneration of seminiferous tubules relative to the chicks intoxicated with Pb (Figure 5). 

**Figure 6:**
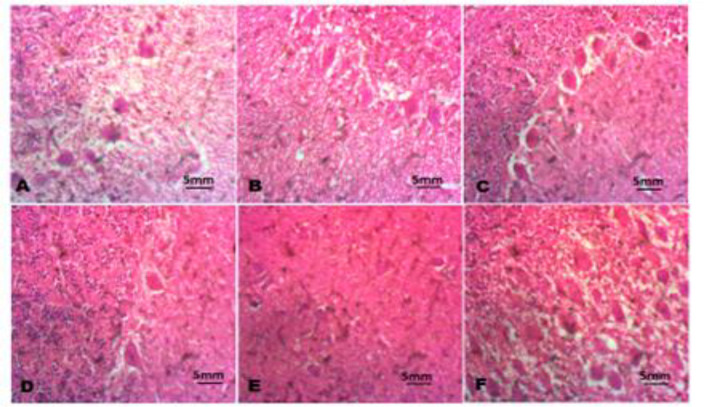
The histopathology of the brain. A (Control), B (Lead acetate; 600 ppm), C (Lead acetate + Naringin 80 mg/kg), D (Lead acetate + Naringin 80 mg/kg), E (Naringin 80 mg/kg), and F (Naringin 100 mg/kg). Slides were stained with Haemtoxylin and Eosin. (Magnification x 400).

NAR in a dose-dependently ameliorated anatomical anarchy precipitated Pb toxicity. Again, we discovered that swelling of neurons with loss of arborization and acute necrosis of neurons in brain of Pb exposed chicks (Figure 6). The neuroprotective effect of NAR was demonstrated with no visible lesions in brain tissues of chicks co-administered with NAR (Figure 6).

**Immunohistochemistry**

**Figure 7a:**
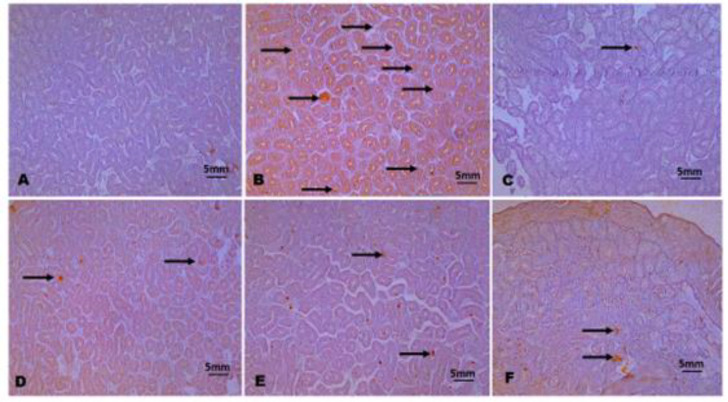
The immunohistochemistry of testicular caspase 3. A (Control), B (Lead acetate; 600 ppm), C (Lead acetate + *Naringin 8*0 mg/kg), D (Lead acetate + Naringin 80 mg/kg), E (Naringin 80 mg/kg), and F (Naringin 100 mg/kg). Slides were stained with high-definition Haemtoxylin. (Magnification x 100).

**Figure 8a:**
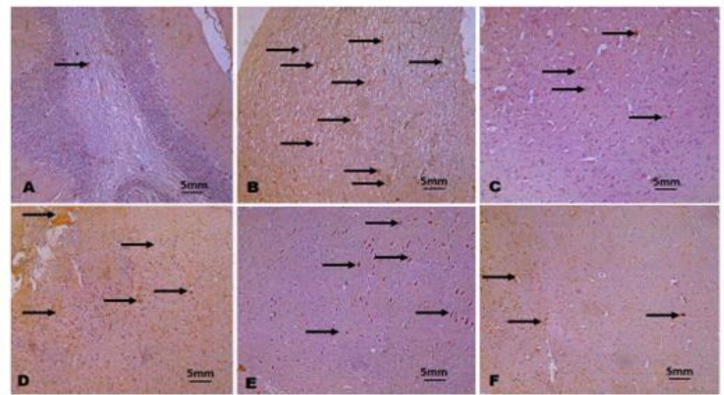
The immunohistochemistry of neuronal Tumor Necrosis Factor alpha (TNF alpha). A (Control), B (Lead acetate; 600 ppm), C (Lead acetate + Naringin 80 mg/kg), D (Lead acetate + Naringin 80 mg/kg), E (Naringin 80 mg/kg), and F (Naringin 100 mg/kg). Slides were stained with high-definition Haematoxylin. (Magnification x 100).

The immuno-localization of testicular caspase 3 and brain TNF-α revealed higher expressions in Pb exposed chicks relative to control and groups co-treated with NAR (Figures 7 and 8). The reduction in the expressions of caspase 3 and TNF-α was indicative of anti-apoptotic, anti-inflammatory, and neuroprotective effects of NAR against Pb acetate-induced testicular cell death and associated neuro-inflammation. 

## Discussion

Pb poisoning has contributed significantly to public health risk, thereby negatively impacting the avian ecosystem health. Pb is a male reproductive toxicant that causes reproductive dysfunction and infertility (Al-Megrin et al., 2020). The testicular cells are easily subjected to oxidative damage due to their high contents of polyunsaturated fatty acids (PUFA) making testicular tissues prone to lipid peroxidation, oxidative stress, and apoptosis (El-khadragy et al., 2020). The pro-apoptotic gene of caspase-3 overexpression in Pb toxicity has been found to stimulate apoptotic processes (Descalzo et al., 2021). In this study, we proposed that Pb intoxication might activate testicular programmed cell death. Pb toxicity has been shown to enhance testicular cell death by raising caspase-3 expression, with concomitant testicular toxicity (Matuq Al-Yasi et al., 2021). However, from our results, NAR co-administration lowered caspase 3 expression. Pb exposure has also been linked to testicular damage and spermatogenic cell death in previous investigation (El-Khadragy et al., 2020). Similarly, Pb administration has been documented to cause exaggerated increase in oxidative stress, inflammation, and apoptosis (Yardim et al., 2020). In the present study, we observed induction of oxidative as indicated with exaggerated raise in MDA content, hydrogen peroxide (H_2_O_2_) generation, and reduction in reduced glutathione content in testes and brain tissues. Oxidative stress is the imbalance between antioxidant and ROS generation in favor of ROS (Ileriturk et al., 2021). Pb toxicity has been reported to cause oxidative stress (Oyem et al., 2021). Furthermore, oxidative stress induction has been associated with heavy metal toxicity and contamination in poultry as described by Touyz et al. (2020). Our findings are consistent with previous reports on oxidative stress, inflammation and apoptosis following Pb intoxication (Wang et al., 2022; Bhattacharya, 2022). The anti-apoptotic and antioxidant property of NAR as recorded in this study could be of a great economic benefit to the global poultry industry. The testicular apoptosis observed in our study was reversed in NAR-treated chicks. Therefore, NAR could be incorporated into poultry feeds to improve reproductive toxicity associated with infertility in breeder poultry farms contaminated with heavy metals such as Pb. Hence, the combination of anti-inflammatory, antioxidant, and metal-chelating properties of NAR could be explored in poultry feeds contaminated with heavy metals.

Pb is one of several neurotoxins that interferes with and diminishes intracellular communication in nerve processes. Neurodegeneration appears to be linked to degenerative alterations and apoptosis in the neural tissue, and this could occur due to ability of Pb to cross the blood brain barrier (Yu et al., 2021). Lead toxicity produces free radicals and causes dysfunctional homeostatic milieu (Gadde and Betharia, 2021). Nerve cells have lower capacity to detoxify ROS, making them prone to increases in ROS levels, which can be caused by Pb poisoning, thereby precipitating neurological processes (Omobowale et al., 2016). From our study, Pb-treated chicks were seen to have marked oxidative stress levels and depletion in the antioxidant defense systems which corroborates other studies (Zhang et al., 2021). However, NAR administration was able to quench oxidative stress indices thereby restoring the antioxidant defense system as indicated in the improvement in *in vivo* antioxidant machinery (Wang et al., 2019). The improvement in antioxidant capacity of NAR can be linked to its observed anti-oxidative and anti-apoptotic properties. Nitric oxide (NO) concentrations in the brain could be used as an indicator of neuronal nitrosative stress (Rafaiee et al., 2021). Without a doubt, NO-induced oxidative imbalance has been reported to trigger inflammatory processes (Oladapo et al., 2021). It is worth noting that previous research has linked NAR's neuroprotective properties to its ability to suppress iNOS expression. Upregulation of NO in the brain has been correlated with neuro-inflammation and oxidative stress as previously described elsewhere (Ajibade and Ogundero, 2021). From our data, co-administration of Pb with NAR significantly attenuated neuro-inflammation induced by Pb intoxication (Umukoro et al., 2018). This therefore supports the reported documentations on the anti-inflammatory action of NAR (Hadjira et al., 2022). Therefore, the present study is in consonance with anti-inflammatory and neuroprotective actions of NAR (Akintunde et al., 2020). We found that Pb exposure caused a considerable increase in the level of TNF-α, as evidenced by immunohistochemistry. The involvement of TNF-α signaling pathway in neuro-inflammation has been reported (Wang et al., 2020). However, we discovered that NAR reversed Pb toxic effects which corroborates the neuroprotective effects of NAR in accordance with other studies (Varshney and Garabadu, 2021). We hypothesized that the anti-inflammatory action of NAR might be through suppression and or mitigation of TNF-α signaling pathway. The link between testicular damage and neuroinflammation as recorded in the present study, might be connected to the hypothalamic-pituitary-gonadal axis. The disruption of this axis by Pb toxicity could be responsible for the brain and testicular damage observed from our findings. Therefore, NAR could open a novel therapeutic window for the management of conditions associated with neuro-inflammation precipitated by Pb toxicity.

It is therefore recommended that NAR could be incorporated into poultry feed as a metal-chelating antioxidant, anti-stress, and heavy metal binder as an alternative method of improving poultry health. The major limitation of this study was inadequate funding precipitated by global COVID-19 pandemic. In the current post-pandemic era, we hope to source for more funds to gain deeper insights into the molecular mechanism of NAR against lead toxicity. This enables us to unravel the mechanism of Pb toxicity and its negative impact on hypothalamic-pituitary-gonadal axis and the reproductive system.

## Conflicts of interest

The authors have declared that there is no conflict of interest.
